# Regulation of zygotic genome activation by the nucleocytoplasmic ratio

**DOI:** 10.3389/fcell.2026.1819263

**Published:** 2026-04-01

**Authors:** Lily Acker, Hui Chen

**Affiliations:** Department of Biological Sciences, University of South Carolina, Columbia, SC, United States

**Keywords:** cell size, early embryogenesis, maternal-to-zygotic transition, nascent transcription, nuclear size, nucleocytoplasmic ratio, spatial pattern, zygotic genome activation

## Abstract

During early development, embryos undergo the maternal-to-zygotic transition (MZT), when control of development shifts from maternal factors to the newly activated zygotic genome. Decades of studies in various model organisms suggest that the nucleocytoplasmic (N/C) ratio is a key regulator of zygotic genome activation (ZGA), though this relationship is more nuanced in some organisms than others. Changing the nuclear content, nucleus size or cell size has been shown to shift the timing of ZGA. Mechanistically, the N/C ratio is linked to fundamental cellular processes that regulate genome activities, including nuclear import, repressor titration, activator accumulation, cell cycle lengthening, and chromatin remodeling. In this review, we summarize the experimental evidence supporting the N/C ratio as a regulator of ZGA and describe the associated molecular mechanisms. We also discuss the limitations of the N/C ratio model, highlight species-specific differences, and examine outstanding questions.

## 1 Introduction

Zygotic genome activation (ZGA) is a defining event in early embryogenesis that transfers developmental control from maternal factors to the embryo’s genome. This process, known as the maternal-to-zygotic transition (MZT), couples the onset of zygotic transcription with the degradation of maternal products. Both maternal clearance and ZGA are essential for early development, supporting embryonic survival, cell fate specification, germ layer formation, and gastrulation (Schier, 2007; Tadros and Lipshitz, 2009; Vastenhouw et al., 2019). Although the timing varies significantly across species, ZGA is conserved across all metazoans, suggesting the involvement of evolutionarily conserved principles alongside species-specific regulatory mechanisms (Lee et al., 2014; Joseph et al., 2017; Palfy et al., 2017; Schulz and Harrison, 2019; Zhou and Heald, 2023).

**FIGURE 1:**
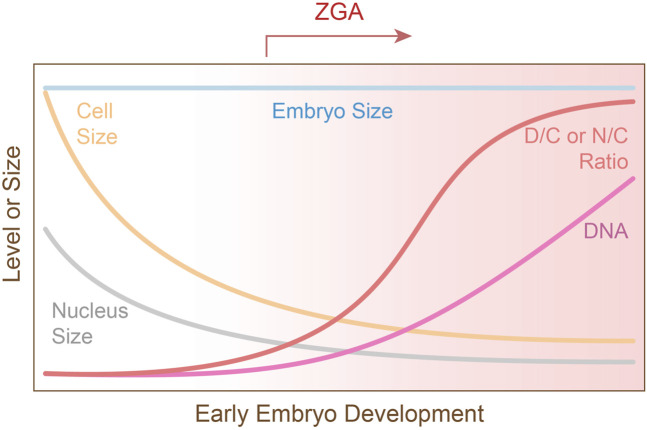
The nucleocytoplasmic (N/C) ratio in early embryogenesis. While early embryo size remains constant, repeated rounds of DNA replication and cell divisions result in exponential increase in DNA quantity, as well as a reduction in cell and nucleus size. This causes an increased DNA-to-cytoplasm (D/C), or nucleocytoplasmic (N/C), ratio which triggers zygotic genome activation (ZGA). The gradient in the background indicates increasing level of zygotic transcription.

**FIGURE 2:**
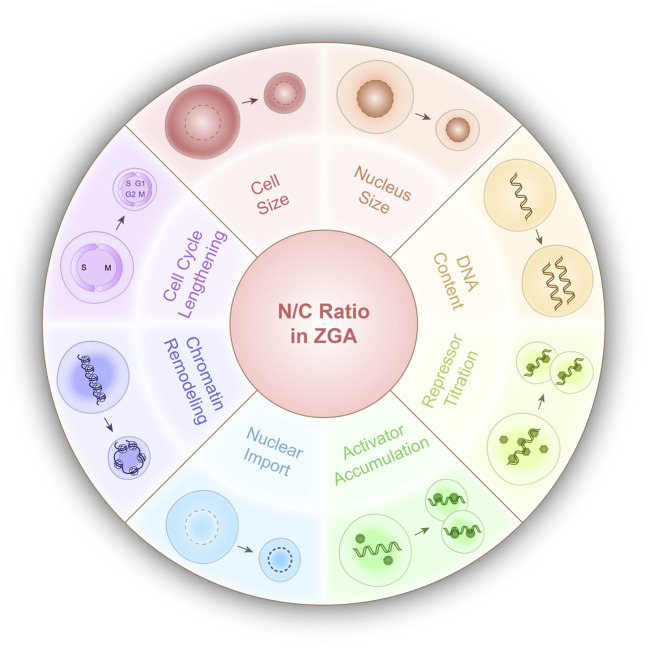
Mechanisms linked to the N/C ratio regulated zygotic genome activation (ZGA). Schematic of the proposed N/C ratio-dependent regulatory mechanisms influencing zygotic genome activation (ZGA). (Top) Unequal reduction of nuclear size and cell size during cleavage division leads to an increase in nuclear-to-cytoplasmic volume ratio. (Right) Increasing DNA content progressively titrates maternally supplied repressors, reducing transcriptional repression during embryogenesis. (Bottom) The coordination of growing nuclear import capacities and activator accumulation further promotes the activation of the zygotic genome. (Left) Systemic alterations to cell cycle length and chromatin accessibility promote a permissive environment for transcriptional activation to occur.

Prior to ZGA, embryos undergo successive rounds of DNA duplication and cell division without growth, exponentially increasing the DNA content and decreasing both the cell size and the nucleus size (Figure 1). This results in an increase in the DNA-to-cytoplasm (D/C) ratio (i.e., DNA content relative to cytoplasmic volume) or the nucleocytoplasmic (N/C) ratio (i.e., nuclear content or volume relative to cytoplasmic volume) (Figure 1). Historically, because the DNA content and the nuclear size are tightly coupled, the D/C ratio and N/C ratio are used interchangeably, except that the D/C ratio is used when DNA content *per se* is the focus (Balachandra et al., 2022). Experimentally manipulating the DNA content, nucleus volume, or cytoplasmic volume alters ZGA timing in various model organisms (Lu et al., 2009; Jevtić and Levy, 2017; Chen et al., 2019; Jukam et al., 2021), supporting the nucleocytoplasmic (N/C ratio) as a conserved regulator of ZGA timing, though the degree of influence can vary among species. This ratio is mechanistically linked to reprogramming processes that establish a permissive environment for ZGA, such as nuclear import, repressor titration, activator accumulation, cell cycle lengthening, and chromatin remodeling (Kojima et al., 2025). Here, we review experimental evidence and mechanisms supporting the N/C ratio as a key regulator of ZGA timing (Figure 2), as well as discuss limitations, species-specific differences, and outstanding questions.

## 2 N/C ratio and ZGA timing at the cellular level

### 2.1 DNA content

Manipulating DNA content has implicated it as a determinant of ZGA in various model organisms. In *Xenopus*, the midblastula transition (MBT) is triggered once a threshold N/C ratio is reached (Newport and Kirschner, 1982a). Additionally, *Xenopus* haploid embryos experience delayed zygotic gene transcription (Jevtić and Levy, 2017), and *Drosophila* haploid embryos have N/C ratio dependent delays in nearly one-third of genes. This N/C ratio in *Drosophila* was shown to directly regulate zygotic transcription through various modalities targeting cell cycle duration, transcriptional activation kinetics, and initiation probability (Syed et al., 2021). Beyond ploidy, experimental strategies have further implicated DNA content in regulating ZGA timing. Increasing genomic DNA by plasmid injection or polyspermy accelerates transcriptional activation (Newport and Kirschner, 1982b; Prioleau et al., 1994). Analyses in *Xenopus* further demonstrated that transcriptional onset scales with the D/C ratio, rather than absolute time. Together, these findings support increasing DNA content, and the subsequent increase in N/C ratio, as regulators of ZGA timing (Jukam et al., 2021).

### 2.2 Nucleus size

During early cleavage divisions, although both nuclear and cell volumes progressively decrease, the reduction in cytoplasm volume outpaces that of the nucleus volume. This causes the N/C ratio to increase prior to ZGA (Jevtić and Levy, 2015). Experimentally increasing nuclear volume in *Xenopus* via microinjecting nuclear scaling factors (importin, lamin) induces premature ZGA, and similarly decreasing nuclear volume by microinjecting reticulon delays zygotic transcription (Jevtić and Levy, 2015). Interestingly, in haploid *Xenopus* embryos, increasing nuclear volume partially restores, while decreasing nuclear volume further delays, zygotic gene transcription (Jevtić and Levy, 2017). These data suggest both nuclear volume and DNA content regulate ZGA timing, two factors highly interconnected with N/C ratio.

### 2.3 Cell size

Due to a lack of single-cell analysis of ZGA, the impact of cell size on ZGA timing was not understood until recently. Metabolic labeling followed by wholemount embryo imaging of the nascent transcriptome in *Xenopus* reveals that large-scale ZGA onset in each cell is dependent on it reaching a threshold size (Chen et al., 2019). This analysis further demonstrated a cell size-dependent spatial pattern corresponding to the endogenous cell size gradient, with small cells at the animal pole initiating ZGA earlier than large cells at the vegetal pole (Chen et al., 2019). Mini-embryos generated by physically reducing the cytoplasmic volume of 1-cell embryos show premature ZGA in a dose-dependent manner (Chen et al., 2019). This suggests that cell size reduction, and subsequent N/C ratio escalation, occurring in early development is sufficient to induce ZGA. Thus, cell size, the denominator of the N/C ratio, is also a critical regulator of ZGA timing.

## 3 Molecular mechanisms linking the N/C ratio to ZGA

### 3.1 Nuclear import

Nuclear conditions are shaped by importing nuclear factors from the cytoplasm, which enter through nuclear pore complexes. However, reduction in cytoplasmic volume during cleavage divisions alters the effective concentration and nuclear import kinetics of maternal factors. In zebrafish, imports through nuclear pore complexes (NPCS) become more efficient as NPCs mature in size and complexity. Genetic mutation of the NPC components impairs nuclear transport of maternal factors and subsequent ZGA onset (Shen et al., 2022). Similarly, perturbing nuclear import dynamics shifts ZGA timing in *Xenopus* (Jevtić and Levy, 2015; evtić and Levy, 2017), indicating that nuclear transport mediates the effects of N/C ratio on ZGA.

Notably, nuclear imports in *Xenopus* are hierarchically organized by their affinity for importins, as factors with higher importin affinity accumulate earlier in the nucleus. This produces temporally ordered changes in nuclear compositions prior to ZGA (Nguyen et al., 2022). Additionally, palmitoylation of importin-α allows it to act as a sensor for cell surface area-to-volume ratio at the plasma membrane, coordinating nuclear transport and scaling intracellular structures to match cell size (Brownlee and Heald, 2019). Global N/C ratio scaling modulates nuclear transport capacity, accumulates key regulators, and partitions maternal factors, thereby creating a permissive environment for ZGA.

### 3.2 Limiting maternal factors

A central model of ZGA timing proposed that maternal repressors are diluted by the increasing DNA content during cleavage. One such repressor is histone proteins, which bind universally across the genome and physically prevent machinery from accessing DNA. For instance, in *Xenopus*, excess histones suppress genome activity through competition between chromatin assembly and transcription complex assembly (Prioleau et al., 1994). Furthermore, egg extract analyses identify histones H3 and H4 as zygotic transcription inhibitors - depleting H3 by DNA beads increases transcription, while adding H3 back restores transcription inhibition (Amodeo et al., 2015). Manipulating free histone abundance similarly alters the timing of specific zygotic gene activation in zebrafish (Joseph et al., 2017). With divisions and rising N/C ratio, the finite maternal histone pool is progressively titrated against exponentially increasing DNA, thereby reducing nucleosome-mediated repression (Amodeo et al., 2015).

DNA replication factors, including RecQ4, Treslin, Cut5, and Drf1 in *Xenopus*, are also titrated maternal factors essential to ZGA (Collart et al., 2013). Unlike histones, which remain relatively constant (Amodeo et al., 2015), these factors become progressively limiting, activating checkpoint kinase Chk1 and lengthening the cell cycle. Conversely, experimentally overexpressing these factors prolongs rapid divisions and increases DNA content after the MBT (Collart et al., 2013). Maternally supplied dNTPs also become limiting, and co-injection with replication factors reduces Chk1 activation (Collart et al., 2013). In *Drosophila,* maternal dNTPs are insufficient for late cleavage DNA replication, requiring *de novo* synthesis. Depletion induces replication stress and checkpoint activation (Song et al., 2017). Together, these data suggest that N/C ratio-dependent titration of maternal factors influence cell cycle slowing and subsequent ZGA.

### 3.3 Transcriptional activators

In contrast to maternally supplied repressors, many transcriptional activators accumulate over time through ongoing translation of maternal mRNAs (Schulz and Harrison, 2019). While activator levels are independent of DNA content, their functional impact depends on chromatin accessibility, which is constrained by histone abundance and overall nuclear composition. Pioneer factors, including Zelda in *Drosophila* (Liang et al., 2008), Pou5f3/SoxB1/Nanog in zebrafish (Lee et al., 2013; Leichsenring et al., 2013), and DUX family proteins and Nr5a2 in mammals (De Iaco et al., 2017; Gassler et al., 2022), bind otherwise inaccessible nucleosome-bound DNA, initiating chromatin opening prior to widespread ZGA (Zaret and Carroll, 2011). As histone-mediated repression is progressively relieved and nuclear import capacity improves, these activators can enter the nucleus and begin transcription. Therefore, ZGA timing reflects the integration of histone titration, ordered nuclear import, and activator accumulation, which all scale with cleavage progression and the N/C ratio.

### 3.4 Cell cycle control

Prior to ZGA, embryos transition from rapid gapless cleavage cycles to longer cycles with gap phases. Due to limited elongation time, short interphase cycles first limit nascent transcription to short, intron-poor genes (Edgar et al., 1986; Heyn et al., 2014; Kwasnieski et al., 2019). Experimentally arresting the embryonic cells at interphase induces precocious ZGA in both *Drosophila* (Strong et al., 2020) and *Xenopus* (Kimelman et al., 1987; Chen and Good, 2022). Further, N/C ratio-dependent mechanisms such as DNA content (Syed et al., 2021), nucleus volume (Jevtić and Levy, 2015), histones (Amodeo et al., 2015), replication factors and dNTPs (Collart et al., 2013) are coupled with alterations to cell cycle length. However, inhibiting zygotic transcription does not prevent cell cycle slowing (Kane and Kimmel, 1993) and arresting cell cycle progression does not necessarily delay ZGA (Zhang et al., 2014). This indicates that transcription and cell cycle remodeling are coupled, but not strictly causally interdependent.

### 3.5 Chromatin remodeling

Chromatin remodeling broadly regulates transcriptional activation through promoting accessibility, and is modulated by multiple mechanisms in ZGA, including histone titration, pioneer factors, and cell cycle slowing. However, chromosome-specific modulations are also crucial to early development. H2A.Z is enriched at up to 65% of zygotically activated genes in *Drosophila* prior to ZGA and RNA Polymerase II loading, and is inferred to be essential for the *de novo* establishment of ZGA transcriptional programs (Ibarra-Morales et al., 2021). Additionally, maternally provided H3.3 variants facilitate minor ZGA by reprogramming parental chromatin in mammals (Zhang et al., 2025), and the local N/C ratio functions as a major determinant of H3.3 incorporation during ZGA in *Drosophila* (Bhatt et al., 2025). Pre-marking H3K4me is required for successful ZGA in *Xenopus* (Oak et al., 2025), and multiple active histone modifications cooperatively shape ZGA programs in non-mammalian vertebrates (Fukushima and Takeda, 2025). Similarly to other factors, an increasing N/C ratio could influence the accessibility and import dynamics of these histone variants and modifications, thereby facilitating ZGA onset.

On a larger scale, the formation of 3D genome architecture influences embryonic success, as the genome is largely unstructured prior to ZGA and thus requires organization to create a transcriptionally permissive environment (Ing-Simmons et al., 2022). Around the time of ZGA, the formation of hierarchical chromatin structures occurs, including topologically associated domains (TADs), loops, fountains and compartments, the dynamics for which are heterogeneous among species (Sivkina et al., 2025). The 3D genome structure is shaped by chromatin factors that are potentially influenced by N/C ratio. Therefore, it would be intriguing to study whether and how the N/C ratio directly contribute to the 3D genome organization in early embryogenesis.

## 4 Conclusion

Cumulative genetic and embryological evidence supports that N/C ratio reprograms the nucleus as a systems-level regulator of ZGA timing. Increasing nuclear content, either by DNA dosage or nuclear volume, advances the onset of transcription and cell cycle remodeling. Mechanistically, rising N/C ratio is proposed to titrate maternally supplied repressors (e.g., histones), promote nuclear import of activators, alter chromatin accessibility, and trigger cell cycle lengthening. Coordinated action of these factors during early development thus creates a permissive environment for zygotic transcription.

### 4.1 Limitations of the N/C ratio model

Despite substantive evidence, the N/C ratio model is not universally predictive, as it regulates specific gene sets rather than globally licensing transcription. In *Drosophila,* haploids initiate large-scale ZGA despite reduced DNA content, and N/C ratio sensitivity is restricted to only a subset of genes (Lu et al., 2009). Similarly, the N/C ratio does not affect transcription globally in mice, although it does induce observable morphological changes (Lee et al., 2001). The influence of N/C ratio on ZGA timing is also species dependent. While organisms with rapid, tightly timed ZGA such as *Xenopus* and *Drosophila* exhibit particularly strong coupling of N/C ratio-dependent mechanisms and ZGA, other organisms with more gradual or multi-phased ZGA, such as zebrafish or mammals, do not show as tight a link to N/C ratio dynamics (Schulz and Harrison, 2019).

Furthermore, the mechanisms discussed here position N/C ratio upstream of proximate transcriptional triggers, such as histone titration, replication stress, activator accumulation, and cell cycle lengthening. Time-dependent maternal programs such as regulated translation, cytoplasmic polyadenylation, and pioneer factor accumulation can drive ZGA when N/C ratio modulations are altered or absent. Together, these findings frame N/C ratio as a quantitative regulator within a broader network, rather than a singular ‘master’ switch.

### 4.2 Outstanding questions

Despite progress in identifying ZGA timing regulators, several key questions remain. It is unclear whether species differences in ZGA onset reflect differential implementation of conserved principles or fundamentally distinct strategies. A quantitative cross-species analysis of the N/C dynamics is needed to establish whether a unified biophysical framework underlies these processes. Furthermore, spatial and temporal ZGA heterogeneity at the single-cell or single-nucleus level has not been fully characterized. Most models treat ZGA timing as uniform; however, local variations in the nuclear composition, chromatin states, replication timing, or maternal factor availability may shift N/C ratio thresholds in individual cells. Single-cell and spatial transcriptomics, combined with live imaging of transcription and chromatin dynamics, will be essential in determining if ZGA timing is globally or locally coordinated.

Finally, the functional importance of N/C ratio-dependent regulation requires characterization beyond early embryogenesis, as it has been implicated in various physiological and pathological conditions. Understanding how N/C ratio-dependent mechanisms operate in early embryos could provide new insight into the general principles of genome regulation, cell fate specification, and disease development.
